# Probable Post-Traumatic Stress Disorder and Associated Factors Among Children Exposed to the 2023 Al Haouz Earthquake in Morocco

**DOI:** 10.3390/healthcare14121787

**Published:** 2026-06-21

**Authors:** Meriyam Hannoun, El Mahjoub El Harsi, Abdelhafid Benkssim, Mohamed Cherkaoui

**Affiliations:** 1 Laboratory of Pharmacology, Neurobiology, Anthropobiology and Environment, Faculty of Sciences Semlalia, Cadi Ayyad University, Marrakech 40000, Morocco; 2 Higher Institute of Nursing Professions and Technical Health (ISPITS-M), Regional Health Directorate, Marrakech 40000, Morocco

**Keywords:** children, earthquake, probable post-traumatic stress disorder, associated factors, Al Haouz, Morocco

## Abstract

**Background/Objectives**: The Al Haouz earthquake that struck Morocco on 8 September 2023, resulted in substantial material, human, and psychological impacts. Children are at increased risk of psychological disorders, notably post-traumatic stress disorder (PTSD). This study aims to assess probable PTSD and its associated factors among children exposed to the Al Haouz earthquake. **Methods**: This cross-sectional study, conducted between December 2024 and January 2025, included 536 children from the affected areas. Probable PTSD was assessed using the 20 item Child and Adolescent Trauma Screen (CATS). Sociodemographic and exposure-related data, including post-earthquake conditions, were collected using a structured questionnaire. Data analysis was performed using SPSS software, version 25. **Results**: Analysis revealed that 47.6% of the children presented with probable PTSD. Multivariable analysis identified several factors independently associated with probable PTSD among children exposed to the earthquake, including age between 11 and 15 years (AOR = 2.02; *p* < 0.001), female gender (AOR = 1.82; *p* = 0.001), advanced level of education (AOR = 1.87; *p* = 0.006), housing damage (AOR = 2.08; *p* = 0.015), physical injury (AOR = 1.86; *p* = 0.012), proximity to the epicenter (AOR = 2.22; *p* = 0.006), temporary shelter in tents (AOR = 1.75; *p* = 0.02), difficulty of evacuation (OR = 1.97; *p* = 0.01), and loss of a family member (AOR = 1.98; *p* = 0.013). **Conclusions**: This study revealed a high frequency of probable PTSD in children exposed to the Al Haouz earthquake and identifies several associated factors, highlighting the need to targeted, multidimensional interventions.

## 1. Introduction

Natural hazard-related disasters in 2023 totaled 399 events, resulting in 86,473 deaths and affecting 93.1 million people worldwide. Economic losses were estimated at USD 202.7 billion [1]. The most devastating event of the year, in terms of both mortality and economic impact, was the earthquake that struck Turkey and the Syrian Arab Republic [2,3].

In addition, two other seismic events were among the 10 deadliest disasters of the year. An earthquake in western Afghanistan caused 2445 deaths [4,5,6]. In Morocco, a 6.8 magnitude earthquake in the High Atlas Mountains, southwest of Marrakech, resulted in 2946 deaths, thousands of injuries, and widespread material damage. Given its substantial human, health, and social impact, this event represents a major public health challenge in Morocco [1,7].

Earthquakes are considered among the most destructive natural disasters that trigger humanitarian crises [8]. They can have adverse effects on the physical and mental health of the affected population, as well as on overall well-being, particularly vulnerable populations such as the elderly, pregnant women, people with chronic illnesses or disabilities, and children [9,10].

Children are particularly vulnerable to the effects of earthquakes due to their developmental stage, their dependence on adults, their limited ability to understand and cope with traumatic experiences. This psychological vulnerability increases their risk of developing mental health disorders, including depression, anxiety and post-traumatic stress disorder [11,12].

Post-traumatic stress disorder (PTSD) is a priority in mental health care following an earthquake, given its high frequency and its persistence in the medium and long term [13]. Previous studies have shown that the prevalence of PTSD in children affected by earthquakes ranges from 4,5% to 95% [14,15].

Post-traumatic stress disorder is a debilitating condition that may develop in individuals and children following exposure to a traumatic event involving a threat of death or serious injury. It has a significant impact on physical, mental and emotional well-being, which can lead to impaired social functioning, emotional distress, and cognitive deficits [16]. This mental disorder manifests in adults and children aged 6 years and older through symptoms of intrusion, avoidance, negative alteration in cognition and mood and changes in arousal and reactivity [17].

Numerous studies have shown that several factors are significantly associated with the development of PTSD in children exposed to earthquakes [18,19]. The most significant Factors may include physical injury, difficulty evacuating during the earthquake, the loss of family member, living in a tent, proximity to the epicenter, damaged housing, age, gender, and low parental education level [20,21,22].

Indeed, it is important to identify at an early stage the children most at risk of PTSD in order to implement the most appropriate post-earthquake psychosocial interventions and mitigate the harmful effects of PTSD [23].

The powerful earthquake that struck the High Atlas Mountains in Morocco on 8 September 2023, had a major multifaceted impact, including loss of life, numerous injuries, destruction of infrastructure, and disruption to healthcare services in the region. Morocco was not adequately prepared for such an event, particularly in these rural areas near the earthquake’s epicenter [24].

The effects of earthquakes on the mental health of Moroccan children are insufficiently documented. Scientific studies should examine the consequences of earthquakes on the psychological health of affected children and the factors associated with them. These investigations are essential to inform the development of contextually appropriate care guidelines, as well as to identify the most significant risk factors for common disorders such as PTSD [25].

In this context, the aim of this study is to estimate the prevalence of probable PTSD among children affected by the Al Haouz earthquake and to identify the factors associated with probable PTSD.

## 2. Materials and Methods

### 2.1. Ethical Considerations

This study was conducted in accordance with the international ethical principles of the World Medical Association Declaration of Helsinki and received approval from the Ethics Committee of the University Hospital of Marrakech (reference No. 99/2024). Written informed consent was obtained from the parents or legal guardians of all participating children. Before data collection, the study objectives and procedures were explained to the children in language appropriate to their age and level of understanding, and verbal assent was obtained from all participants. Children presenting scores suggestive of probable PTSD were confidentially referred to their parents or legal guardians and advised to seek further psychological assessment. When necessary, participants were directed to the nearest primary healthcare facility and referred through the Moroccan healthcare system for appropriate mental health care. Given the vulnerable status of the participating children, collaboration with local healthcare structures was established to ensure ethical conduct of the study and facilitate timely access to appropriate support services for children experiencing probable PTSD symptoms The anonymity and confidentiality of participants’ data were strictly maintained throughout the research process, and participation was entirely voluntary.

### 2.2. Study Design

This research was designed as an analytical cross-sectional study conducted 15 months after the Al Haouz earthquake in Morocco. It focused on children aged 7 to 15 years attending primary schools in rural areas most affected by the 8 September 2023, earthquake in Al Haouz province. This age group was selected as it corresponds to a school-aged population that is easily accessible in the post-earthquake context. Children in this age group also possess the cognitive abilities to reliably respond to assessment tools and self-report symptoms. Furthermore, this age group is particularly vulnerable to PTSD, with a high prevalence reported in the literature [26,27,28].

Data collection was conducted 15 months after the earthquake. This timing was influenced by several preparatory requirements, including ethical approval, administrative authorizations, parental consent procedures, and coordination with schools located in remote rural and mountainous areas affected by the disaster. Furthermore, the post-earthquake context was characterized by considerable logistical challenges and disruptions to educational activities.

In addition, assessing children beyond the immediate post-disaster period allowed the evaluation of the medium-term psychological impact of the earthquake. Similar assessment timeframes have been reported in previous studies among child earthquake survivors, including evaluations conducted 15 months and up to 36 months after the disaster [20].

Following the Al Haouz earthquake, the educational sector was severely affected. According to UNESCO [29], the earthquake impacted a predominantly rural and isolated area comprising approximately one million students and more than 42,000 education professionals, with over 500 schools and around 50 boarding schools reported as damaged. In Al Haouz Province, educational statistics for the 2023–2024 academic year indicated the presence of 184 primary schools, including 169 located in rural areas [30]. Given the extent of the damage to educational infrastructure, many educational activities were temporarily relocated to provisional learning facilities.

A single-stage probability cluster sampling method was used, with schools serving as the sampling clusters. Primary schools closest to the earthquake epicenter were randomly selected, and all eligible students within the selected schools were invited to participate. This methodological choice was justified by its operational feasibility and suitability to the post-seismic context, characterized by significant logistical constraints and difficulties in accessing the child population. This process was carried out after obtaining authorization from the regional directorate of the Ministry of National Education, Preschool, and Sports in Al Haouz Province.

The required sample size was calculated prior to data collection. Based on the estimated number of eligible children enrolled in each school and considering the logistical constraints of conducting research in a post-earthquake context, six schools were considered sufficient to achieve the minimum required sample size. These schools were randomly selected from the eligible schools located in the areas most affected by the earthquake and closest to the epicenter. Random selection was used to enhance the representativeness of the sample and improve the generalizability of the findings. All children meeting the inclusion criteria within the selected schools were invited to participate, and all eligible students were included in the study, resulting in a final sample size slightly exceeding the minimum required sample size.

Using Schwartz’s standard formula for estimating proportions, *n*= z^2^ × *p* (1 − *p*)/d^2^, the minimum sample size required for this study was calculated. With *n* = minimum sample size, z = 1.96 (value for the 95% confidence interval), *p* = 0.5, and d = margin of error (0.05). Given the substantial heterogeneity of PTSD prevalence estimates reported among children exposed to earthquakes, ranging from 4.5% to 95% across studies [15,16], and the absence of a single estimate that could be considered sufficiently representative of the target population, a prevalence of 50% was used for sample size calculation. This conservative approach is consistent with the recommendations of Lwanga and Lemeshow [31], who indicate that a value of 50% represents the most conservative choice because it yields the maximum required sample size and minimizes the risk of underestimating the study population. After adjusting for a 10% non-response rate or incomplete questionnaires, considering the unstable post-earthquake school environment, the minimum required sample size was estimated at 427 participants. A total of 536 children were included in the study.

This study aimed to investigate probable PTSD and its associated factors among children exposed to the Al Haouz earthquake. It was conducted among children attending six randomly selected primary schools located in the most affected rural communities. At the time of data collection, educational activities in these schools were being conducted in temporary learning structures established following earthquake-related damage to school infrastructure. Healthcare professionals specifically trained in data collection procedures and the administration of assessment instruments conducted the survey. The inclusion criteria were children who attended the selected primary schools, aged 7 to 15 years and were present in the affected region at the time of the earthquake. The exclusion criteria were a history of psychiatric disorders or severe cognitive or neurological conditions pre-existing before the seismic event that could influence the understanding of the assessment tool’s questions.

### 2.3. Data Collection

Data were collected using structured questionnaires at the selected primary schools, in quiet rooms that provided appropriate conditions for conducting the survey. Healthcare professionals trained in mental health were responsible for administering the questionnaires, which were administered with assistance when needed to the participating children. The questionnaires were well-structured and included both closed-ended and multiple choice questions. Each questionnaire comprised three sections. The first section of the form contained questions about the various sociodemographic characteristics of the children and their families. The second section focused on data regarding the children’s exposure and experiences during the earthquake and their post-earthquake living conditions. The third section was designed to assess probable PTSD symptoms using the child and Adolescent Trauma Screen (CATS).

### 2.4. Variables and Methods

Data were collected using a questionnaire that included sociodemographic characteristics, information related to the extent of exposure to the earthquake, and an assessment of probable PTSD symptoms. The sociodemographic variables examined were age, gender, grade level, place of residence, parent’s marital status, family structure, and parent’s educational level.

The variables related to exposure to the traumatic event and the post-earthquake context that were explored included housing damage, physical injury, proximity of the epicenter, current housing situation after the earthquake, location at the time of the seismic event, difficulty of evacuation, and loss of a family member.

The Child and Adolescent Trauma Screen (CATS) is an internationally validated, brief, and freely available questionnaire intended to screen for exposure to potentially traumatic events and symptoms of probable PTSD. The CATS is designed to assess symptoms of PTSD based on the Diagnostic and Statistical Manual of Mental Disorders –Fifth Edition (DSM-5) criteria for PTSD in young people aged 7–17 years. Its items are directly related to the criteria for intrusion, avoidance, negative alterations in cognition and mood, and hyper arousal. Participants first select the traumatic events they have experienced from a list of 15 items, then identify the most distressing event. They then rate 20 items on a four-point Likert scale ranging (from 0 = “never” to 3 = “almost always”). The total score ranges from 0 to 60, and a score of 21 or higher indicates probable PTSD [32].

The CATS is an instrument that has proven its reliability and validity across diverse cultural contexts and has been validated and translated into multiple languages. The availability of an Arabic translation makes it possible to use the tool among Arabic-speaking populations and it is can be accessed through academic platforms [33,34,35].

### 2.5. Statistical Analysis

Data were analyzed using IBM SPSS software, version 25. Normality of continuous variables was assessed using the one-sample Kolmogorov–Smirnov test. Quantitative variables were described using means and standard deviations, and qualitative variables were presented as frequencies and percentages. Chi-square tests were performed to identify the association between the various categorical variables and probable PTSD. Variables associated with probable PTSD in the bivariate analysis at a significance level of *p* < 0.20 were entered simultaneously into a multivariable logistic regression model using the Enter method to identify factors independently associated with probable PTSD and to control for potential confounding factors. Probable PTSD status (yes/no) was used as the dependent variable. The significance of individual variables was assessed using the Wald chi-square test. The strength of associations between probable PTSD and related factors was estimated using adjusted odds ratios (AORs) with 95% confidence intervals (CIs). Model performance was evaluated using the Hosmer–Lemeshow goodness-of-fit test, Nagelkerke R^2^, classification accuracy, and multicollinearity diagnostics (tolerance and variance inflation factor). A *p*-value of 0.05 or less was considered statistically significant.

## 3. Results

### 3.1. Sociodemographic Characteristics

As shown in Table 1, the sociodemographic characteristics of children exposed to the earthquake are presented.

A total of 536 participants were included in the study, with a mean age of 9.59 years (SD = 2.04). The majority of children were aged between 7 and 10 years (74.8%), while only 25.2% were aged between 11 and 15 years, with a predominance of boys compared to girls. Participants’ grade levels ranged from first to sixth grade and were relatively evenly distributed. Six rural communes affected by the earthquake were represented in the sample, with the highest proportion of participants from Talat N’Yacoub (21.6%). Most children lived with both parents (93.7%), and their parents’ educational attainment was generally low.

### 3.2. Sociodemographic Factors Associated with Probable PTSD

The association between probable PTSD and sociodemographic factors is presented in Table 2.

The bivariate analysis showed that among the 536 participating children, 255 (47.6%) had probable PTSD, while 281 (52.4%) did not. Age was significantly associated with probable PTSD (*p* < 0.001), with a higher proportion among children aged 11 to 15 years (61.5%) than among those aged 7 to 10 years (42.9%). Gender was also significantly associated with probable PTSD (*p* = 0.001), with a higher proportion of girls among participants with probable PTSD (52.6%) compared to boys (43.7%). While boys predominated among those without probable PTSD. Furthermore, the children’s grade level was significantly associated with probable PTSD (*p* < 0.001). The proportion of children in higher-grade levels was greater among participants with probable PTSD (56.8%) compared to those in lower grade levels (36.2%). In contrast, family structure, parental education were not significantly associated with probable PTSD.

### 3.3. Factors Related to Traumatic Exposure and Post-Earthquake Context Associated with Probable PTSD

As shown in Table 3, the association between probable PTSD and earthquake exposure and post-disaster context factors was examined.

Several variables were significantly associated with probable PTSD. Probable PTSD was more common among children whose homes were damaged (50.9%) compared to those whose homes were not damaged (28.2%) (*p* < 0.001). Similarly, a higher proportion of probable PTSD was observed among participants with physical injuries (65.8%) compared to uninjured participants (42.5%) (*p* < 0.001). Proximity to the earthquake epicenter showed a statistically significant association with probable PTSD (*p* = 0.002), with higher rates among children living in areas closer to the epicenter. In the same way, the type of housing after the seismic event was significantly associated with probable PTSD (*p* = 0.001), with a higher proportion of participants with probable PTSD among children living in tents (48.9%) compared to those living in houses (36.7%). Difficulty of evacuation during the earthquake was also strongly associated with probable PTSD (*p* < 0.001). Probable PTSD was commonly reported participants who were trapped at the time of the earthquake (57.5%), in comparison to children who did not experience evacuation difficulties (39.5%). In addition, loss of a family member was significantly associated with probable PTSD (*p* = 0.002). Probable PTSD was commonly reported among children who lost a family member (49.1%) than among those who did not experience a family loss (47.1%). However, the location at the time of the earthquake was not significantly associated with probable PTSD.

### 3.4. Multivariate Analysis

The logistic regression analysis identified several factors independently associated with probable PTSD after adjustment for potential confounders (Table 4).

Prior to model construction, multicollinearity was assessed, and no evidence of problematic collinearity was detected, with tolerance values ranging from 0.759 to 0.978 and VIF values ranging from 1.018 to 1.119. The final multivariable logistic regression model demonstrated an adequate fit to the data, as indicated by the Hosmer–Lemeshow goodness-of-fit test (χ^2^ = 12.51, *p* = 0.130). The model explained 26.7% of the variance in probable PTSD (Nagelkerke R^2^ =0.267) and correctly classified 75.9% of participants.

Concerning sociodemographic variables, age was identified as significantly associated with probable PTSD. Children aged 11–15 years had 2.02-fold-higher odds of probable PTSD compared to those aged 7–10 years (AOR = 2.02; 95% CI: 1.09–3.05; *p* < 0.001). Additionally, female gender was independently associated with probable PTSD, with girls having 1.82-fold-higher odds of probable PTSD than boys (AOR = 1.82; 95% CI: 1.11–2.92; *p* = 0.001). Children’s grade level was also significantly associated with probable PTSD, compared with children in lower grade levels, those in higher grade levels had greater odds of probable PTSD (AOR = 1.87; 95% CI: 1.2–2.91; *p* = 0.006).

Regarding variables related to earthquake exposure and the post-disaster context, several factors were significantly associated with probable PTSD. Housing damage was significantly associated with probable PTSD, with children whose homes were damaged having 2.08-fold-higher odds of probable PTSD than those whose homes were undamaged (AOR = 2.08; 95% CI: 1.15–3.77; *p* = 0.015). Similarly, physical injury was significantly associated with probable PTSD, with injured children having 1.86-fold-higher odds of probable PTSD compared to uninjured children (AOR = 1.86; 95% CI: 1.15–3.02; *p* = 0.012). Proximity to the earthquake epicenter was also significantly associated with probable PTSD, with children living in areas closest to the epicenter having 2.22-fold-higher odds of probable PTSD than those residing in more distant areas (AOR = 2.22; 95% CI: 1.25–3.93; *p* = 0.006).

In addition, post-earthquake housing conditions were significantly associated with probable PTSD, with children living in tents having higher odds of probable PTSD compared to those living in houses (AOR = 1.75; 95% CI: 1.10–3.18; *p* = 0.02). Difficulty of evacuation during the earthquake was also significantly associated with probable PTSD, with children who experienced evacuation difficulties having approximately twofold-higher odds of probable PTSD than those who did not experience such difficulties (AOR = 1.97; 95% CI: 1.33–3.66; *p* = 0.01). Furthermore, loss of a family member was significantly associated with probable PTSD, with children who experienced such loss having 1.98-fold-higher odds of probable PTSD than those who did not (AOR = 1.98; 95% CI: 1.14–3.46; *p* = 0.013). Certain variables, although significant in bivariate analysis, did not retain their significance after multivariable adjustment, notably location at the time of the earthquake and maternal educational level.

## 4. Discussion

This study aimed to estimate the prevalence of probable PTSD 15 months after the Al Haouz earthquake among exposed children and to identify associated factors.

The results revealed a high prevalence of probable PTSD among children exposed to the Al Haouz earthquake (47.6%), substantially exceeding that reported in the general child population, estimated at approximately 2.17% over their lifetime. This prevalence also surpasses rates reported among children exposed to various traumatic events, including violence, natural disasters, and abuse, which are generally estimated at around 25% [36,37]. This disparity underscores the magnitude of the psychological impact of this seismic disaster on children. Moreover, the observed prevalence is consistent with findings from the international literature on seismic disasters, where several studies have reported comparable or even higher PTSD rates among exposed children [20,38]. However, lower prevalence rates have also been some populations [39,40]. More broadly, estimates of PTSD prevalence among children exposed to earthquakes vary widely, ranging from 4.5% to 95% across studies [15,16]. This variability can be attributed to differences in individual characteristics, the intensity and nature of exposure to the trauma, and post-disaster conditions [2,19,41].

The findings of this study identified several factors independently associated with probable PTSD, suggesting that children exposed to the earthquake are particularly vulnerable. Among individual and sociodemographic factors, age emerged as a factor significantly associated with probable PTSD, with older children being more likely to present probable PTSD. School grade, which is closely related to age, was also identified as a significant factor associated with probable PTSD. These findings are consistent with several previous studies and may be explained by more developed cognitive abilities, enabling older children to better understand the severity of the traumatic event, anticipate its consequences, and retain more persistent traumatic memories. School grade therefore appears to be a contributing, rather than a central, factor associated with probable PTSD in children [19,42,43,44,45]. Additionally, female gender was identified as a factor significantly associated with probable PTSD, as girls were more likely to present probable PTSD following exposure to traumatic events such as earthquakes. This result is in accordance with the literature, suggesting that this specific vulnerability may stem from heightened emotional reactivity in girls, combined with a greater tendency toward internalizing coping strategies, particularly rumination, which may contribute to the persistence of post-traumatic symptoms, as well as the impact of contextual characteristics of the exposure [23,27,46,47]. Previous research has also suggested that girls may be more psychologically vulnerable to post-traumatic stress reactions. Furthermore, socialization processes may influence how children perceive, express, and cope with emotional distress. Girls are generally encouraged to recognize and express their emotions, whereas boys are more often encouraged to control or suppress them. These differences in psychological vulnerability, emotional expression, and coping responses may partly explain the higher prevalence of probable PTSD observed among girls following natural disasters [48].

Other factors related to direct traumatic exposure to the earthquake and its aftermath were also found to be significantly associated with probable PTSD. Housing damage and physical injury emerged as factors significantly associated with probable PTSD, corroborating previous studies [11,19]. Probable PTSD was more frequent among children who were injured during the earthquake, suggesting that physical injury may serve as an indicator of trauma severity. This association may be explained by the combination of direct exposure to a life-threatening situation and the experience of physical pain [38]. Similarly, housing loss was associated with probable PTSD, which may be explained by the intensity of traumatic exposure resulting from home destruction and the disruption of the living environment [49]. In line with previous studies, proximity to the earthquake epicenter was significantly associated with probable PTSD. Children living in the areas closest to the earthquake epicenter were more likely to present probable PTSD. This finding may be explained by the higher level of exposure in these areas, notably the extent of human and material losses and the heightened perception of life-threatening danger. These areas were generally the most severely affected [50,51,52,53].

Furthermore, children living in tent camps were more likely to present probable PTSD. These findings are consistent with those reported in other post-earthquake studies, which have documented a positive association between precarious housing conditions and PTSD among children [38,54]. Post-earthquake living conditions, particularly residence in temporary shelters different from the original home environment, may serve as a constant reminder of the traumatic event, reactivating traumatic memories and contributing to the persistence of post-traumatic symptoms [55]. Evacuation difficulties were also significantly associated with probable PTSD, in line with previous research. Being trapped under rubble can be considered a severe traumatic experience involving intense fear and an imminent threat to life, both of which have been associated with post-traumatic stress symptoms [43,51,56,57]. Likewise, loss of a family member was significantly associated with probable PTSD. The literature consistently shows that children who have lost a loved one are more likely to experience post-traumatic stress symptoms following traumatic events. This association may be explained by direct exposure to death and the disruption of attachment bonds, both of which may increase psychological vulnerability [19,58]. The grieving process may further intensify post-traumatic symptoms, particularly in the presence of intense emotional responses, difficulties in emotion regulation, and maladaptive coping strategies [59,60]. Moreover, exposure to loss in a traumatic context may contribute to persistent psychological distress, especially in cases of complicated grief [61].

Some variables, particularly maternal educational level and the child’s location at the time of the earthquake, were significantly associated with probable PTSD in the bivariate analysis but lost significance after adjustment in the multivariable model. This finding suggests that their apparent associations may be explained by other factors more directly related to trauma exposure and post-disaster living conditions. Maternal educational level may reflect broader socioeconomic vulnerabilities that indirectly influence children’s mental health through family resources, access to psychological support, and post-disaster living circumstances [11,62]. Likewise, the child’s location during the earthquake may represent an indirect indicator of the traumatic experience rather than an independently associated factor. Evidence indicates that subjective aspects of trauma exposure, including perceived threat, intense fear, a sense of danger to life, and evacuation difficulties, are more strongly associated with post-traumatic stress outcomes than certain demographic or socioeconomic characteristics [43,63]. Furthermore, long-term psychological outcomes among earthquake-exposed children appear to be more closely related to the severity of exposure, stressful life events, and family psychological factors than to isolated sociodemographic characteristics [52]. Therefore, the loss of significance observed after adjustment may reflect confounding effects or indirect pathways through which these variables are associated with probable PTSD via factors more strongly related to trauma severity and post-disaster adversities [37]. However, given the cross-sectional nature of the present study, mediation mechanisms cannot be formally established and should be further explored in longitudinal research [64].

Beyond individual characteristics and disaster exposure, probable PTSD among children may also be influenced by pre-existing social vulnerabilities. Factors such as poverty, socioeconomic disadvantage, rural residence, educational inequalities, and limited access to mental health and psychosocial services may hinder recovery following a disaster. These disadvantages can reduce access to support services and community resources, thereby increasing the likelihood of persistent psychological distress. In addition, children from low-income families are often more exposed to secondary adversities following a disaster, such as financial difficulties and social isolation, which may further complicate the recovery process [65]. This highlights the importance of considering how disaster exposure intersects with broader social determinants of health when examining children’s post-disaster mental health outcomes [66,67].

The results of the present study also provide a relevant framework for guiding intervention strategies. An integrated approach combining early identification of high-risk children, longitudinal follow-up, targeted psychosocial interventions, psychoeducation, and tailored family support may help mitigate the effects of trauma, prevent symptom chronicity, promote resilience, and improve the overall well-being of affected children.

### Strengths and Limitations

Despite the relevance of the findings, this study has some limitations. Data collection relied partly on self-reported measures, which may be subject to reporting bias. The study was conducted 15 months after the earthquake, which may have introduced recall bias regarding earthquake-related experiences. In addition, probable PTSD symptoms may fluctuate over time, and the findings may not fully reflect symptom patterns during earlier stages of post-disaster recovery. Furthermore, the cross-sectional design, although appropriate for assessment in the post-disaster context, does not allow for the examination of symptom trajectories over time or the establishment of causal relationships. The study included only school-attending children from rural areas heavily affected by the earthquake. Therefore, the findings may not fully represent all children affected by the disaster, particularly those who were not attending school. Consequently, caution is warranted when generalizing the findings beyond the study population, as non-school-attending children may have experienced different levels of vulnerability and post-disaster adversity. Future longitudinal studies and intervention-based research are needed to better understand the evolution of PTSD symptoms and to inform strategies aimed at reducing their impact among affected children.

Nevertheless, this study has several important strengths. It focuses on a particularly vulnerable and un-der-documented population, specifically children exposed to the Al Haouz earthquake in Morocco, within a resource-limited setting. The comprehensive assessment of sociodemographic characteristics, trauma-related variables, and post-disaster conditions enabled the identification of key determinants of PTSD, providing a more integrated understanding of the phenomenon. These findings may offer valuable insights into the development of targeted and contextually appropriate intervention strategies.

## 5. Conclusions

Beyond loss of life and material damage, earthquakes can have profound and long-lasting psychological consequences, particularly among children. This study revealed a high rate of probable PTSD among children exposed to the Al Haouz earthquake and identified several associated factors. The results showed that individual variables, such as age, gender, and grade level, as well as factors related to direct traumatic exposure and post-earthquake conditions, including housing damage, physical injury, proximity to the epicenter, living in tents, evacuation difficulties, and the loss of a family member, were significantly associated with probable PTSD.

These findings highlight the substantial psychological burden experienced by children following the Al Haouz earthquake and underscore the importance of considering both disaster-related exposures and broader psychosocial vulnerabilities when assessing post-traumatic outcomes. Early identification and continued monitoring of vulnerable children remain essential to support recovery and reduce the long-term consequences of post-traumatic stress.

## 6. Implications for Practice and Policy

The findings of the present study have important implications for mental health practice, school-based interventions, and disaster response policies. An integrated approach combining early identification of vulnerable children, longitudinal follow-up, and targeted psychosocial interventions may contribute to reducing the long-term impact of trauma and promoting resilience among affected children. The development of psychoeducational interventions in schools, especially those incorporating play-based and participatory approaches, may help create safe environments, enhance psychosocial skills, promote emotional regulation, and prevent long-term mental health problems.

In addition, play therapy represents a promising preventive and therapeutic approach, as it has been shown to reduce post-traumatic stress symptoms among children who have experienced an earthquake [68]. As a simple, accessible, and effective intervention, it is well suited to post-disaster contexts where specialized mental health resources are limited and can be applied in both schools and community settings.

## Figures and Tables

**Table 1:** Sociodemographic characteristics of children exposed to the earthquake.

Variable	Frequency (*n*)	Percentage (%)
**Age (years), mean ± SD**	9.59 ± 2.04	
7–10 years	401	74.8
11–15 years	135	25.2
**Gender**		
Male	302	56.3
Female	234	43.7
**School level**		
1st year of primary education	83	15.5
2nd year of primary education	65	12.1
3rd year of primary education	92	17.2
4th year of primary education	96	17.9
5th year of primary education	90	16.8
6th year of primary education	110	20.5
**Place of residence**		
Anougal	104	19.4
Azgour	103	19.2
Ighil	70	13.1
Talat N’Yacoub	116	21.6
Imgdal	49	9.1
Asni	94	17.5
**Parental marital status**		
Married	506	94.4
Divorced	8	1.5
Mother deceased	2	0.4
Father deceased	19	3.5
Other family situation	1	0.2
**Family structure**		
Living with both parents	502	93.7
Living with father only	2	0.4
Living with mother only	28	5.2
Living with other family members	4	0.7
**Father’s educational grade**		
No formal education	155	28.9
Primary education	287	53.5
Secondary education	82	15.3
Higher education	12	2.3
**Mother’s educational grade**		
No formal education	226	42.2
Primary education	277	51.7
Secondary education	25	4.7
Higher education	8	1.4

Abbreviations: *n*, number of participants; SD, standard deviation.

**Table 2:** Association between sociodemographic factors and probable PTSD among child survivors.

Variable	Probable PTSD (Yes) *n* (%)	Probable PTSD (No) *n* (%)	χ2	*p*-Value
Age			13.99	<0.001
7–10 years	172 (42.9)	229 (57.1)		
11–15 years	83 (61.5)	52 (38.5)		
Gender			10.34	0.001
Male	132 (43.7)	170 (56.3)		
Female	123 (52.6)	111 (47.4)		
Educational level			22.35	<0.001
Basic cycle (grades 1–3)	87 (36.2)	153 (63.7)		
Intermediate cycle (grades 4–6)	168 (56.8)	128 (43.2)		
Family structure			1.13	0.260
Living with both parents	242 (48.2)	260 (51.8)		
Not living with both parents	13 (38.2)	21 (61.8)		
Father’s educational grade			0.02	0.952
Low	202 (47.5)	223 (52.5)		
Medium to high	45 (47.9)	49 (52.1)		
Mother’s educational grade			2.79	0.095
Low	242 (48.3)	259 (51.7)		
Medium to high	11 (33.3)	22 (66.7)		

Abbreviations: probable PTSD, probable post-traumatic stress disorder; *n*, number of participants; χ2, Pearson’s chi-square test.

**Table 3:** Factors related to traumatic exposure and post-earthquake context associated with probable PTSD among child survivors.

Variable	Probable PTSD (Yes) *n* (%)	Probable PTSD (No) *n* (%)	χ2	*p*-Value
Housing damage			13.73	<0.001
Yes	233 (50.9)	225 (49.1)		
No	22 (28.2)	56 (71.8)		
Physical injury			19.96	<0.001
Yes	77 (65.8)	40 (34.2)		
No	178 (42.5)	241 (57.5)		
Distance to epicenter			12.87	0.002
Very close (<10 km)	81 (46.8)	92 (53.2)		
Close (11–30 km)	142 (52.8)	127 (45.2)		
Distant (>30 km)	32 (34)	62 (66)		
Current housing situation			10.34	0.001
House	22 (36.7)	38 (63.3)		
Tent	233 (48.9)	243 (51.1)		
Location at the time of the earthquake			3.84	0.050
Outside the house	235 (47.3)	262 (52.7)		
Inside the house	20 (51.3)	19 (48.7)		
Difficulty evacuating			17.17	<0.001
Yes	138 (57.5)	102 (42.5)		
No	117 (39.5)	179 (60.5)		
Loss of a family member			9.56	0.002
Yes	57 (49.1)	59 (50.9)		
No	198 (4.1)	222 (52.9)		

Abbreviations: probable PTSD, probable post-traumatic stress disorder; *n*, number of participants; χ2, Pearson’s chi-square test.

**Table 4:** Factors independently associated with probable PTSD based on multivariable logistic regression Analysis.

Variable	β (Coefficient)	Wald	AOR (95% CI)	*p*-Value
Age (11–15 years)	0.704	14.023	2.023 (1.09–3.05)	<0.001
Gender (Female)	0.599	7.234	1.819 (1.11–2.92)	0.007
Mother’s educational grade	0.556	1.826	1.744 (0.79–3.91)	0.177
Housing damage	0.734	5.874	2.084 (1.15–3.77)	0.015
Physical injury	0.621	6.281	1.860 (1.15–3.02)	0.012
Distance to epicenter				
Distant > 30 km (Reference)				
Close 11–30 km	0.368	1.372	1.445 (0.78–2,67	0.241
Very close < 10 km	0.797	7.483	2.219 (1.25–3.93)	0.006
Current housing (tent)	0.561	5.415	1.752 (1.10–3.18)	0.02
Location at the time of the earthquake	−0.388	1.293	0.679 (0.35–1.33)	0.256
Difficulty evacuating	0.760	5.967	1.968 (1.33–3.66)	0.01
Loss of family member	0.923	6.153	1.981 (1.14–3.46)	0.013
Children’s educational level (advanced)	0.625	7.606	1.869 (1.2–2.91)	0.006

Abbreviations: β, regression coefficient; AOR, adjusted odds ratio; CI, confidence interval; *p*, *p*-value.

## Data Availability

The data are not publicly available due to ethical considerations, as the study involves children and includes sensitive personal information. Therefore, the data are available from the corresponding author upon reasonable request.
